# Telehealth Uptake Among Hispanic People During COVID-19: Retrospective Observational Study

**DOI:** 10.2196/57717

**Published:** 2024-07-24

**Authors:** Di Shang, Cynthia Williams, Hera Culiqi

**Affiliations:** 1 University of North Florida, Jacksonville, FL, United States; 2 University of Central Florida, Orlando, FL, United States

**Keywords:** telehealth, telemedicine, ICT, eHealth, e-health, Hispanic, health equity, health access, Hispanics, digital divide, usage, utilization, equity, inequity, inequities, access, accessibility, Spanish, observational, demographic, demographics, socioeconomic, socioeconomics, information and communication technology

## Abstract

**Background:**

The Hispanic community represents a sizeable community that experiences inequities in the US health care system. As the system has moved toward digital health platforms, evaluating the potential impact on Hispanic communities is critical.

**Objective:**

The study aimed to investigate demographic, socioeconomic, and behavioral factors contributing to low telehealth use in Hispanic communities.

**Methods:**

We used a retrospective observation study design to examine the study objectives. The COVID-19 Research Database Consortium provided the Analytics IQ PeopleCore consumer data and Office Alley claims data. The study period was from March 2020 to April 2021. Multiple logistic regression was used to determine the odds of using telehealth services.

**Results:**

We examined 3,478,287 unique Hispanic patients, 16.6% (577,396) of whom used telehealth. Results suggested that patients aged between 18 and 44 years were more likely to use telehealth (odds ratio [OR] 1.07, 95% CI 1.05-1.1; *P*<.001) than patients aged older than 65 years. Across all age groups, patients with high incomes were at least 20% more likely to use telehealth than patients with lower incomes (*P*<.001); patients who had a primary care physician (*P*=.01), exhibited high medical usage (*P*<.001), or were interested in exercise (*P*=.03) were more likely to use telehealth; patients who had unhealthy behaviors such as smoking and alcohol consumption were less likely to use telehealth (*P*<.001). Male patients were less likely than female patients to use telehealth among patients aged 65 years and older (OR 0.94, 95% CI 0.93-0.95; *P*<.001), while male patients aged between 18 and 44 years were more likely to use telehealth (OR 1.05, 95% CI 1.03-1.07; *P*<.001). Among patients younger than 65 years, full-time employment was positively associated with telehealth use (*P*<.001). Patients aged between 18 and 44 years with high school or less education were 2% less likely to use telehealth (OR 0.98, 95% CI 0.97-0.99; *P*=.005). Results also revealed a positive association with using WebMD (WebMD LLC) among patients aged older than 44 years (*P*<.001), while there was a negative association with electronic prescriptions among those who were aged between 18 and 44 years (*P*=.009) and aged between 45 and 64 years (*P*=.004).

**Conclusions:**

This study demonstrates that telehealth use among Hispanic communities is dependent upon factors such as age, gender, education, socioeconomic status, current health care engagement, and health behaviors. To address these challenges, we advocate for interdisciplinary approaches that involve medical professionals, insurance providers, and community-based services actively engaging with Hispanic communities and promoting telehealth use. We propose the following recommendations: enhance access to health insurance, improve access to primary care providers, and allocate fiscal and educational resources to support telehealth use. As telehealth increasingly shapes health care delivery, it is vital for professionals to facilitate the use of all available avenues for accessing care.

## Introduction

Health inequities among people of racial and ethnic minority groups are a significant concern across the United States of America. The Hispanic or Latino (hereafter “Hispanic”) population is a community that experiences inequities in access to care. However, health inequities in the United States of America are primarily compared between non-Hispanic White and non-Hispanic Black populations, with some positioning of the Hispanic population. Therefore, we must highlight the needs of the Hispanic community as a matter of priority. A previous study suggested that health inequities between non-Hispanic Black and Hispanic people are similar in health risks and outcomes [1]. Boen and Hummer [1] attributed much of the similarities to the influence of socioeconomic status and stress in these communities. Social determinants of health significantly impact the health and wellness of people of color. In addition, the inequity in health outcomes is confounded by challenges in access to care. Gaining access to and using health care services is essential to mitigating adverse health outcomes and promoting equity in quality care [2]. Low access to health care services is due to a complexity of factors, which include a lack of insurance, education, and fiscal resources [2]. Non-Hispanic White women and men experienced greater usage of care, 83% and 74%, respectively; among Hispanic individuals, women had higher usage rates than men, 68% and 50%, respectively; however, they still fall short of their non-Hispanic White counterparts [2].

While there are considerable efforts to decrease inequities in health care access, recent events such as the proliferation of technology in health care and the global COVID-19 pandemic eclipse equity in access efforts. COVID-19 accelerated the use of telehealth, which was essential to access health care information and services [3]. Telehealth was highly encouraged to mitigate the ill effects of the contagion and support lockdown measures. While other racial and ethnic communities experienced telehealth growth, the Hispanic population experienced relatively lower amounts of telehealth increases and had a disproportioned rise in the use of health care resources [4-7]. When compared to non-Hispanic White individuals, Hispanic patients had lower usage of telehealth services (27% and 6.8%, respectively) [8]. Telehealth usage among Hispanic people fell below that of their non-Hispanic Black counterparts. Hispanic people showed lower adjusted odds of using telehealth when compared to non-Hispanic Black persons before and during the pandemic [9,10]. However, Hispanic patients and people in low-income positions had increased levels of telehealth use during the pandemic [11]. People who experience inequities in access are more likely to have unmet health care needs and have a higher prevalence of morbidity and mortality rates [12]. The lack of telehealth usage was a critical issue during this time. Qian et al [13] found higher rates of COVID-19 cases in regions with low telehealth use. Given the lack of telehealth usage and reduced access to in-person services during the pandemic, it is reasonable to assume that overall health outcomes were compromised.

Research suggested that telehealth use differed by demographics such as gender, age, and education [14]. Studies have noted gender differences in telehealth use, as male participants are less likely to use telehealth than female participants [3,9,15,16]. Among racial and ethnic groups, Hispanic patients often opt for in-person rather than telehealth visits [5,10]. Whether a patient has access to and knowledge to use telehealth depends on socioeconomic factors [5]. Ramirez et al [5] observed that the Hispanic community had lower telehealth use due to cultural perceptions, inadequate financial resources, and digital literacy barriers. Health inequities are concerning, as research suggests that health outcomes are favorable across racial and ethnic groups when services are used [17,18]. The persistence of inequity in health care suggests that access to care is complex and should consider economic and social factors. To effectively incorporate factors influencing care, we comprehensively assess the components contributing to lower usage among Hispanic people. This study aims to examine the socioeconomic, demographic, and behavioral factors that influence telehealth use among people who identify as Hispanic.

## Methods

### Data Source

The study period was from March 2020 to April 2021. The COVID-19 Research Database Consortium provided access to the Office Ally and Analytics IQ PeopleCore consumer databases. The Office Ally database provided access to US claims data from 100 million unique patients and 3.4 billion medical claims. The Analytics IQ PeopleCore consumer database provides individual-level data across demographics, behaviors, and economic indicators and is a national representation of 242.5 million US adults aged 19 years and older. We joined the Office Ally claims data with Analytics IQ PeopleCore consumer data through an identifier, which enabled us to retrieve patient claims data during the study period and examine telehealth usage from socioeconomic and behavioral perspectives, as shown in the “Results” section.

### Ethical Considerations

The COVID-19 Research Database was established by complying with regulatory standards to protect patient privacy. The COVID-19 Research Database received a waiver of patient consent certified from the Western Institutional Review Board for using HIPAA (Health Insurance Portability and Accountability Act)–certified deidentified data on April 20, 2020 [19]. The Western Institutional Review Board granted exemption status for HIPAA-limited data sets and non-HIPAA–covered data on May 14, 2020. This exemption covers all research performed in the COVID-19 Research Database. In addition, researchers with approved study proposals are granted access only to specific data sets necessary to answer their research question or questions. Only deidentified and limited data sets are made available through the database and certified before access is granted. Individual project institutional board approval was optional.

## Results

### Overview

We retrieved 16.43 million Office Ally claim records of Hispanic patients during the study period, and 3,478,287 unique Hispanic patients were included to investigate telehealth usage. A descriptive summary of the patients included in the analyses is in Table 1. Telehealth claims were identified by screening for the procedure modifier codes 95, GT, and GQ. Among the patients, 16.6% (578,945/3,478,287) had one or more telehealth claims during the study period. Female patients had slightly higher telehealth usage (338,795/1,958,350, 17.3%) than male patients (240,150/1,519,937, 15.8%).

**Table 1.** Characteristics of participants in the study (N=3,478,287).

Characteristics		Value
**Sex, n (%)**		
	Female	1,958,350 (56.3)
	Male	1,519,937 (43.7)
**Age group (years), n (%)**		
	18-44	1,133,417 (32.6)
	45-64	1,326,391 (38.1)
	65+	1,018,479 (29.3)
**Education qualification, n (%)**		
	Bachelor or higher	922,435 (26.5)
	High school or less	2,555,852 (73.5)
**Employment status, n (%)**		
	Unemployed	1,227,541 (35.3)
	Part-time	634, 818 (18.2)
	Full-time	1,615,928 (46.5)
**Household annual income (US $), n (%)**		
	Low: <46,000 (1st quartile)	941,294 (27.1)
	Medium: 46,000-144,000	1,670,747 (48)
	High: >114,000 (4th quartile)	866,246 (24.9)
**Having a primary care doctor, n (%)**		
	Yes	2,782,591 (80)
	No	695,696 (20)
**Exhibit high medical use, n (%)**		
	Yes	671,349 (19.3)
	No	2,806,938 (80.7)
**Interest in exercise, n (%)**		
	Yes	700,691 (20.1)
	No	2,777,596 (79.9)
**Measure of alcohol consumption, n (%)**		
	No consumption	353,921 (10.2)
	Some consumption	2,855,276 (82.1)
	High consumption	269,090 (7.7)
**Frequency of smoking, n (%)**		
	Never	413,982 (11.9)
	Some	2,919,447 (83.9)
	Daily	144,858 (4.2)
**Using electronic prescription services, n (%)**		
	Yes	578,768 (16.6)
	No	2,899,519 (83.4)
**Using WebMD, n (%)**		
	Yes	828,260 (23.8)
	No	2,650,027 (76.2)
Total number of claims during the study period, mean (SD)		4.73 (4.71)

### Logistic Regression Analysis

The data were aggregated at the patient level to investigate telehealth use and determine whether a patient used telehealth during the study period. A patient with one or more telehealth claims during the study period was assigned a value of 1 for the dependent variable; otherwise, the value was assigned as 0. Categorical variables were created to stratify patients into groups by their demographics and socioeconomic status, as listed in Table 1. We conducted a multiple logistic regression to determine the odds of patients using telehealth. Each patient’s total number of claims during the study period was included as an offset variable in the logistic regression to control its potential impact on the dependent variable. Results suggested that compared to patients aged older than 65 years, patients aged between 18 and 44 years are 1.07 times (odds ratio [OR] 1.07, 95% CI 1.05-1.1; *P*<.001) likely to use telehealth, while patients aged between 45 and 64 years showed a nonsignificant difference (*P*=.49).

Results from our logistic regression analysis of the 3 age groups are shown in Table 2. Male patients in the older group (aged older than 65 years) are 6% less likely to use telehealth (OR 0.94, 95% CI 0.93-0.95; *P*<.001), while male patients in the young group (aged between 18 and 44 years) are 5% more likely to use telehealth (OR 1.05, 95% CI 1.03-1.07; *P*<.001). Patients with a primary care doctor (*P*=.01) or high medical usage (*P*<.001) are significantly more likely to use telehealth, especially in the patients aged older than 65 years group. Patients who use WebMD (WebMD LLC) are significantly (*P*<.001) more likely to use telehealth among those who are aged older than 44 years. In comparison, the negative association with electronic prescriptions is significant among those who are aged between 18 and 44 years (*P*=.009) and aged between 45 and 64 years (*P*=.004). Patients aged between 18 and 44 years with high school or less education are 2% less likely to use telehealth (OR 0.98, 95% CI 0.97-0.99; *P*=.005). Patients with high incomes across all age groups were more likely to use telehealth than patients with lower incomes, as follows: aged between 18 and 44 years (OR 1.25, 95% CI 1.23-1.28; *P*<.001), aged between 45 and 64 years (OR 1.33, 95% CI 1.3-1.35; *P*<.001), and aged older than 65 years (OR 1.2, 95% CI 1.18-1.22; *P*<.001). Patients younger than 65 years with full-time employment (*P*<.001) are significantly more likely to use telehealth.

Patients with unhealthy behaviors such as alcohol use and smoking are significantly less likely to use telehealth (*P*<.001). In the patients older than 65 years group, patients with high alcohol consumption are 39% less likely to use telehealth than patients with no alcohol consumption (OR 0.61, 95%CI 0.56-0.65; *P*<.001). Patients aged 65 years and older who smoke daily are 36% less likely to use telehealth than patients who never smoke (OR 0.64, 95% CI 0.6-0.69; *P*<.001). Meanwhile, patients interested in exercise were significantly more likely to use telehealth (*P*=.03).

**Table 2.** Odds ratios from logistic regression analysis by age groups.

Variables		Aged 65+ years	Aged between 45 and 64 years	Aged between 18 and 44 years
**Sex: male (reference: female)**				
	Odds ratio (95% CI)	0.94 (0.93-0.95)	0.99 (0.98-1.01)	1.05 (1.03-1.07)
	*P* value	<.001	.35	<.001
**Primary care doctor (reference: no)**				
	Odds ratio (95% CI)	1.19 (1.1-1.29)	1.02 (1-1.04)	1.06 (1.05-1.08)
	*P* value	.001	.01	<.001
**Medical use (reference: no)**				
	Odds ratio (95% CI)	1.09 (1.07-1.1)	1.07 (1.06-1.08)	1.03 (1.02-1.05)
	*P* value	<.001	<.001	<.001
**WebMD (reference: no)**				
	Odds ratio (95% CI)	1.07 (1.04-1.1)	1.05 (1.04-1.07)	1.01 (0.99-1.02)
	*P* value	<.001	<.001	.32
**Electronic prescriptions (reference: no)**				
	Odds ratio (95% CI)	1 (0.99-1.02)	0.97 (0.95-0.99)	0.93 (0.87-0.98)
	*P* value	.46	.004	.009
**Education: high school or less (reference: bachelor or higher)**				
	Odds ratio (95% CI)	1.01 (0.98-1.04)	1 (0.99-1.01)	0.98 (0.97-0.99)
	*P* value	.34	.61	.005
**Employment: full-time (reference: unemployed)**				
	Odds ratio (95% CI)	1.02 (0.97-1.07)	1.07 (1.06-1.09)	1.05 (1.02-1.08)
	*P* value	.40	<.001	<.001
**Income: high (reference: low)**				
	Odds ratio (95% CI)	1.25 (1.23-1.28)	1.33 (1.3-1.35)	1.2 (1.18-1.22)
	*P* value	<.001	<.001	<.001
**Exercise fan: yes (reference: no)**				
	Odds ratio (95% CI)	1.04 (1-1.08)	1.05 (1.03-1.07)	1.03 (1.02-1.05)
	*P* value	.03	<.001	<.001
**Alcohol: high consumption (reference: no consumption)**				
	Odds ratio (95% CI)	0.61 (0.56-0.65)	0.79 (0.77-0.82)	0.81 (0.78-0.85)
	*P* value	<.001	<.001	<.001
**Smoking: daily (reference: never)**				
	Odds ratio (95% CI)	0.64 (0.6-0.69)	0.85 (0.82-0.88)	0.93 (0.89-0.97)
	*P* value	<.001	<.001	<.001

## Discussion

### Overview

This study found that among Hispanic people, male participants aged between 18 and 44 years were more likely to use telehealth than female participants, but male participants aged older than 44 years were less likely to use telehealth than female participants. Across all age groups, people with high incomes, people with primary care physicians, current users of the health care system, people who used WebMD, and people who reported full-time employment were more likely to use telehealth. Patients aged between 18 and 44 years with high school or less education were 2% less likely to use telehealth. There was a negative association with electronic prescriptions among those aged between 18 and 44 years and aged between 45 and 64 years. In addition, regardless of age, people with unhealthy behaviors, such as smoking, alcohol consumption, and a lack of interest in exercise, were less likely to use telehealth services.

### Telehealth and Demographic Factors

The presented analyses represent factors contributing to telehealth use among Hispanic people. Like other studies, people aged older than 65 years were less likely to use telehealth than people in younger age groups [3,9]. Male participants in the aged older than 65 years group were 6% less likely to use telehealth, while male participants in the youngest group were 5% more likely to use telehealth. Saeed and Masters [16] indicated that female participants have higher telehealth usage due to caregiver burdens that make attending in-person visits more challenging. Furthermore, our findings indicate that the positive influence of health care use factors (eg, having a primary care doctor, medical usage, and use of WebMD) and the detrimental effects of unhealthy behaviors (alcohol consumption and smoking) are more pronounced among patients aged older than 65 years compared to younger age groups. Our findings suggest the necessity of considering various age groups when examining usage differences between age and gender groups.

### Telehealth and Socioeconomic Factors

This study’s results on educational background align with other studies that suggested that people with more than a high school diploma have higher telehealth usage [18]. A lack of education contributes to low health and digital literacy and interferes with a person’s inability to access and use health-related information [20,21]. This study suggests that people with a primary care physician and those using the health care system (medical usage) are more likely to use telehealth [9,22]. These characteristics describe people who already have access to the health care system and are perhaps representative of the segment of the Hispanic population that experiences more health-related equity [23]. This has implications for increasing convenient access to care rather than increasing access for people who do not already have access to care. Research suggested that a primary care physician was critical to accessing traditional, in-person services. Having access to a primary care physician provides an avenue to get telehealth services; therefore, we can reasonably speculate that people who have access to in-person health care will have access to telehealth [16,24]. People who live in low-income communities are particularly vulnerable due to a lack of resources [14]. Darrat et al [15] suggested that among people with incomes less than US $30,000 annually, 29% lacked a smartphone, 44% did not have home broadband access, and 46% did not own a computer. The lack of internet access exacerbates the inequity in service access [18]. Jain et al [25] suggested that 84% of telehealth users had broadband internet access [26]. A study by Chau et al [27] indicated that 30% of Hispanic people do not have a computer in their home and are 10 years behind non-Hispanic White people with regard to broadband internet access. Researchers suggested that cultural perspectives influence technology use even when Hispanic individuals have similar technologies [2,26,28]. The results of this study indicate that Hispanic persons who used the internet for health information, such as WebMD, were more likely to use telehealth services; however, there was no relationship with electronic prescription behavior. Haun et al [29] also suggested no statistically significant relationship exists between telehealth use and electronic prescription behavior, as the provider, not the patient, initiates this service. Among Hispanic patients who used telehealth, they had the highest rate of missed telehealth visits at 42% [3]. A study by Ghaddar et al [6] suggested that 60% of Hispanic individuals access the internet or send or receive emails. However, only 24% communicate electronically with health care providers, and 40% report having low digital literacy [6].

### Telehealth and Health Behaviors

In this study, people with unhealthy behaviors, such as smoking, alcohol consumption, and a lack of interest in exercise, had significantly lower odds of using telehealth across all age groups. Researchers suggested that smoking disproportionately affects people of low income and educational status, and alcohol misuse is increasing among people of color and people older than 60 years [30,31]. Health behaviors often include factors such as smoking, drinking, and physical activity and contribute to health inequity [32,33]. In addition, health behaviors have a significant effect on health care usage [34,35]. The association between telehealth use and smoking and alcohol is not conclusive in the body of literature. Jaffe et al [36] suggested that smoking has no relationship with exercise behavior, alcohol use, smoking, or telehealth. They suggested that 12% of people who smoked had a telehealth visit, as compared to 61% of people who never smoked and had a telehealth visit; however, it was not statistically significant (*P*=.45) [36]. The authors also suggested that alcohol consumption was not a deterrent to telehealth use, as 69% of alcohol users experienced a telehealth visit. A study by Wegermann et al [3] indicated that there were minimal to no differences in telehealth use among people who reported alcohol use or smoking. However, another study by Kim [37] indicated that exercise and alcohol use were associated with telehealth acceptance, whereas smoking status was not. A study by Jagielo et al [38] noted that during the COVID-19 pandemic, the stay-at-home order was more predictive of telehealth use than race or ethnicity among smokers. Among people with alcohol use disorders, researchers reported no differences in preference for telehealth or in-person treatment [39,40]. While beyond the scope of this study, it is imperative to note the rapid usage of telehealth and other digital technologies for smoking and alcohol cessation programs and exercise promotion. Given the aforementioned socioeconomic status of people who engaged in unhealthy behaviors, it is not surprising that challenges were noted in access to technology, digital literacy, and quiet session locations [30].

### Recommendations

Glasgow et al [41] suggested that socioeconomic factors, cultural perceptions, and patient preferences significantly impact health care use. By considering patient health behaviors and preferences, providers and decision-makers can support “individual health and public health through enhanced care” [41] and gain a comprehensive understanding of the complex factors contributing to low telehealth access. We make several recommendations based on the results of this and other studies. First, primary care providers serve as the point of care and cost-efficient entry into the health care system [42]. Primary care supports access to other physician specialties, and its services are relatively more amenable to telehealth compared to other physician specialties (42% and 35%, respectively); 73% of primary care services could be offered through telehealth [43]. Community-based primary care clinics can close the health equity gap [44]. Primary care clinics are uniquely positioned to engage communities in the sociocontent of their environment and culture. Using this strategy, local clinics can promote culturally relevant educational strategies and encourage positive behavior change for health outcomes. Second, as we consider supporting telehealth in primary care, it is imperative that we support a digital public health infrastructure. Primary care providers serving people vulnerable to health inequities report that the lack of digital access remains a barrier. Chang et al [14] suggested that primary care providers report that as much as 70% of their patients lack digital or internet access, and 50% are uncomfortable with the technology. Digital access provides a gateway to education and employment; thus, with effective interventions, we can mitigate this social determinant of health [45]. This suggests that discussions about including digital access in the public health infrastructure and as a social determinant of health warrant priority consideration [46].

### Limitations

This study used a unique population-based data source. This allowed us to examine the Hispanic population in the Office Ally and Analytics IQ databases. We studied socioeconomic, health behavior, and demographic factors in the Hispanic community and determined the odds of using telehealth during a public health crisis. The results may not be reproducible, as the data collection was during COVID-19, and patients may not have gained proficiency with the technology or lack internet access. The study used the COVID-19 Research Database and is subject to the limitations of administrative databases. The validity of the data is dependant upon the facilities to report accurate data and code visits correctly in the Office Ally database. Analytics IQ PeopleCore consumer data rely on the accuracy of reporting by the consumer. In this study, the Hispanic population considered all persons of Hispanic or Latino identification. In addition, data were not available on geography (rural and urban), access to home internet, and the ability to read and write English proficiently. In future research, it is imperative to account for variances in usage patterns between urban and rural populations, given the potential impacts of geographic disparities, technological infrastructure, and internet accessibility on health care usage. Future studies should consider measures of English health literacy and its association with health care access. Furthermore, future studies should consider patient and provider relationships in local communities to explore additional information not captured in surveys and claims data that explicates attitudes and challenges with telehealth access and use.

### Conclusions

Telehealth supports favorable health outcomes across populations. However, without equity in usage, these benefits are not realized across communities. This study highlights that telehealth use among Hispanic communities can be influenced by demographic, socioeconomic, and health behavioral factors. Telehealth use among Hispanic communities is dependent upon several important factors, such as age, gender, education, socioeconomic status, current health care engagement, and health behaviors. To overcome these barriers, we recommend interdisciplinary strategies that call for medical professionals, insurance providers, and community-based services to engage meaningfully with Hispanic communities to support telehealth use. As telehealth becomes increasingly prevalent in our society, it is imperative that we support this method for accessing the health care system.

## Abbreviations

HIPAA: Health Insurance Portability and Accountability Act
OR: odds ratio
